# P05.72. Survey to assess the feasibility of providing a brief yoga skills training to improve outcomes of gynecologic cancer surgery

**DOI:** 10.1186/1472-6882-12-S1-P432

**Published:** 2012-06-12

**Authors:** S Sohl, K Stanbery, J Tooze, S Lentz, K Stephenson, S Danhauer

**Affiliations:** 1 Wake Forest School of Medicine, Winston-Salem, USA

## Purpose

More than 65,000 women are diagnosed with gynecologic cancers (i.e., ovarian, endometrial) in the United States each year. Exploratory laparotomy is a common primary treatment for these diagnoses; however, it leads to pain and distress in a majority of patients. Yoga is a promising intervention for improving these negative surgical outcomes. The purpose of this study was to explore the interest in and feasibility of implementing a Yoga Skills Training (YST) for these women.

## Methods

Adults scheduled for an exploratory laparotomy for a suspected gynecologic malignancy were eligible. Screening logs were documented and women were approached while in the hospital prior to and following surgery. Patients enrolled in the study completed questions regarding their interest in the YST, outcome expectations and were asked open-ended questions to clarify responses.

## Results

Of 19 women approached, 9 agreed to complete the survey and one of these was a screen fail. Participants (n=8; mean age=48.5 years [SD=13.8]; 13% non-White) indicated that prayer (75%), gentle movement (75%), relaxation (75%), breathing (63%), counseling (63%), and information (38%) would be helpful to reduce bother they experienced during their hospital stay. A majority of participants (63%) would have been interested in the described YST before and after surgery, although 50% indicated they did not like the name “YST”. Outcome expectations indicated that participants thought a brief YST would be helpful (on a scale from 0-10) for reducing pain (M=6.3[SD=2.3] and distress (M=6.5[SD=1.8]).

## Conclusion

This study showed that there was interest in the YST among women undergoing surgery for a suspected gynecological malignancy. Thus, future studies should implement yoga for these women and investigate its efficacy for reducing negative surgical outcomes. Results also suggested that such future studies recruit before surgery, rename the intervention, and that prayer or counseling are of more interest for a control group than information.

